# Left Atrial Strain as an Integrative Marker of Diastolic Dysfunction and Atrial Fibrillation in Hypertrophic Cardiomyopathy: A Retrospective Cross-Sectional Study

**DOI:** 10.3390/medicina62091787

**Published:** 2026-09-16

**Authors:** Eglė Tamulėnaitė-Stuokė, Joana Ažukaitė, Marius Šukys, Eglė Ereminienė

**Affiliations:** 1 Heart Centre, Medical Academy, Lithuanian University of Health Sciences, LT-44307 Kaunas, Lithuania; 2 Lithuanian Society of Cardiology, LT-50009 Kaunas, Lithuania; 3 Department of Genetics and Molecular Medicine, Medical Academy, Lithuanian University of Health Sciences, LT-50161 Kaunas, Lithuania; 4 Laboratory of Clinical Cardiology, Institute of Cardiology, Lithuanian University of Health Sciences, LT-50162 Kaunas, Lithuania

**Keywords:** hypertrophic cardiomyopathy, speckle-tracking echocardiography, left atrial strain, diastolic dysfunction, atrial fibrillation

## Abstract

*Background and Objectives*: Echocardiographic assessment of left ventricular diastolic dysfunction (LVDD) in hypertrophic cardiomyopathy (HCM) is challenging, as conventional parameters correlate poorly with left ventricular filling pressure. Emerging evidence suggests that left atrial (LA) strain is associated with atrial fibrillation (AF). This study aimed to compare LA reservoir strain (LASr)-based and conventional LVDD grading, evaluate associations between LA strain and clinical and echocardiographic parameters, and explore its relationship with AF. *Materials and Methods*: This retrospective cross-sectional single-center study included 109 adult HCM patients with comprehensive echocardiographic data. LVDD was graded using conventional echocardiographic parameters and LASr-based thresholds (≥35% (grade 0), ≥24% to <35% (grade 1), ≥19% to <24% (grade 2), and <19% (grade 3)). *Results:* LASr-based grading revealed a significantly greater prevalence of advanced LVDD compared with conventional assessment (grade 3: 39% vs. 3%, *p* < 0.001), with poor intermethod agreement (κ = 0.07). Overall, approximately 45% of patients were reclassified into higher LVDD grades using LASr, particularly among nonobstructive HCM patients. Higher LASr-based LVDD grades were associated with worse conventional diastolic indices, reduced biventricular strain parameters, and higher natriuretic peptide levels. Reduced LA reservoir and conduit strain were independently associated with documented AF after adjustment for age, LA volume index, and left ventricular mass index. *Conclusions*: LASr provides additional information on diastolic dysfunction in HCM patients and is associated with documented AF. LASr-based grading assigned more patients to advanced LVDD grades than conventional assessment. The observed association with AF requires prospective validation before LA strain can be used for risk stratification.

## 1. Introduction

Hypertrophic cardiomyopathy (HCM) is the most common inherited heart disease, with a prevalence ranging from 1:200 to 1:500 [1,2]. HCM is primarily inherited in an autosomal dominant pattern (40 to 60% of HCM patients have a single variant as a cause of their disease) [1]. According to the newest European Society of Cardiology (ESC) Guidelines for the management of cardiomyopathies, HCM is defined as increased left ventricle (LV) thickness (with or without right ventricle (RV) hypertrophy) or a mass that is not explained by abnormal loading conditions [1]. Among patients with HCM, 30% to 40% experience adverse events such as sudden cardiac death (SCD), progressive limiting symptoms because of LV outflow tract (LVOT) obstruction or LV diastolic dysfunction (LVDD), heart failure (HF) due to systolic dysfunction, and atrial fibrillation (AF) with a risk of thromboembolic events [2]. The risk of SCD in HCM has decreased significantly, as advances in SCD risk stratification help identify patients at greatest risk who can then undergo implantable cardioverter-defibrillator (ICD) placement. Thus, the focus has shifted to HF as the predominant cause of morbidity and mortality in patients with HCM [2]. HCM is associated with LVDD caused by multifactorial abnormalities, including high intracavity pressure, altered ventricular contraction and relaxation, interstitial fibrosis, and myocardial ischemia [2]. Symptomatic nonobstructive HCM patients present a diagnostic and therapeutic challenge; moreover, their overall risk of HCM-related death is similar to that of patients with obstructive HCM [3]. To achieve optimal medical therapy for LVDD, accurate classification of LVDD severity in patients with HCM is essential. However, this remains challenging in HCM, as many standard echocardiographic parameters have weak correlations with LV filling pressures due to LVOT obstruction and mitral regurgitation (MR), which are common in HCM patients [4]. The recent European Association of Cardiovascular Imaging (EACVI) clinical consensus statement of multimodality imaging in HCM patients proposes an integrated four-criteria approach to assess high LV filling pressures in HCM patients, whereas left atrial reservoir strain (LASr) is recommended in inconclusive cases [5]. Recent data have shown that LASr can also be used for prognostic assessment in patients with HCM, as a higher LASr-based LVDD grade is associated with increased rates of HF-related death or hospitalization [6]. Moreover, LASr has been shown to predict the development of new-onset AF and major adverse cardiac events (MACE) [7].

The aim of our study was to compare LASr-based and conventional diastolic dysfunction grading, evaluate the associations of left atrial (LA) strain with clinical and echocardiographic parameters, and explore its relationship with AF.

## 2. Materials and Methods

### 2.1. Patient Population

Patients ≥ 18 years of age with a diagnosis of HCM who had both echocardiography and genetic testing performed between 1 January 2019 and 15 April 2025 at the Hospital of the Lithuanian University of Health Sciences Kauno Klinikos were screened for inclusion. A total of 129 patients were selected, and 109 were ultimately included in our retrospective study. The exclusion criteria were: patients under 18 years of age, LV hypertrophy due to other causes, no echocardiography performed before septal reduction therapy, and insufficient echocardiographic image quality for measuring LA strain or echocardiographic images not available in the database. Echocardiographic images were unavailable in the database for 7 patients, and in 13 patients the quality of the images available in the database was insufficient for left atrial strain measurement.

Baseline characteristics, including age, sex, body mass index, New York Heart Association (NYHA) functional class, arrhythmias, comorbidities, medication use, presence of ICD, applied HCM septal reduction therapies (myectomy/ablation), B-type natriuretic peptide (BNP) level, and HCM genetic testing, were extracted from the electronic patient information system. AF was defined as previously documented AF in the medical record, including paroxysmal, persistent, or permanent AF form.

The study was approved by the Bioethics Centre of the Lithuanian University of Health Sciences (No. BE-2-63). All included patients had previously provided written informed consent for the use of their anonymized clinical data in scientific research.

### 2.2. Echocardiographic Analysis

Two-dimensional (2D) echocardiography was performed by an experienced echocardiographer using a conventional transthoracic 2D echocardiography system (EPIQ 7, Philips Ultrasound, Inc., Bothell, WA, USA) with a 1.5–4.6 MHz transducer. Stored echocardiographic images were analyzed offline (TomTec-Arena TTA 2.30 Imaging Systems, Unterschleissheim, Germany).

All standard echocardiographic measurements were obtained according to existing recommendations [8]. LV diameter and septal LV wall thickness were calculated from the parasternal long axis view, perpendicular to the long axis of the LV, and measured at the level of the mitral valve (MV) leaflet tips. The maximal LV end-diastolic wall thickness was assessed from the short-axis view at the basal, middle, and apical levels. The LV ejection fraction (LVEF) was calculated using the modified Simpson’s biplane method. The RV dimensions were estimated from an RV-focused apical four-chamber view. The LA diameter was calculated in the parasternal long-axis view. The LA volume was obtained using area length method from the apical four-chamber view at the end of systole. Diastolic parameters (including the mitral inflow early diastolic velocity (E), ratio of early to late mitral inflow velocities (E/A), early diastolic mitral annular velocity (e′), and ratio of the mitral inflow early diastolic velocity to the early diastolic mitral annular velocity (E/e′)) were assessed using Doppler at the tips of the mitral leaflets and from tissue Doppler imaging at the level of the MV annulus. The peak velocity of tricuspid regurgitation (TR) was evaluated by continuous-wave (CW) Doppler [8,9]. LV intraventricular or LVOT obstruction was explored by pulsed wave Doppler and quantified by CW Doppler at rest and during the Valsalva maneuver. In symptomatic patients with rest or provoked LVOT gradient < 50 mmHg, bicycle stress echocardiography was performed. Obstructive HCM was diagnosed when the maximal LVOT gradient was ≥50 mmHg [1].

**Figure 1:**
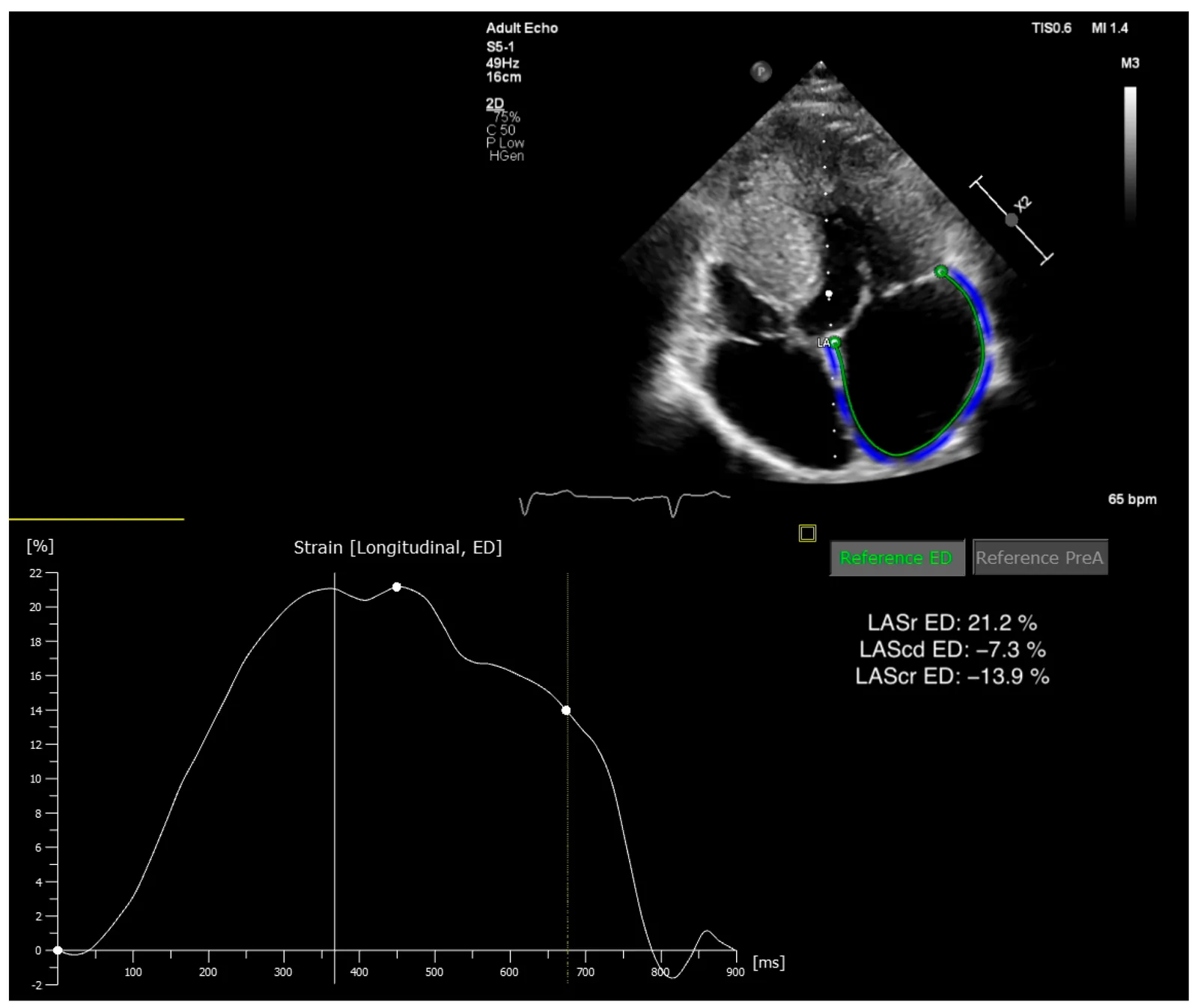
Example of left atrial strain analysis showing reservoir (LASr), conduit (LAScd), and contractile (LASct) strain measurement. Abbreviations: ED, end-diastole.

Myocardial strain was assessed using speckle-tracking echocardiography. For the evaluation of LV global longitudinal strain (LV GLS), apical four-chamber, two-chamber, and three-chamber views were obtained according to existing guidelines [8]. Right ventricular myocardial deformation parameters, including RV free-wall longitudinal strain (RV FWLS) and RV global longitudinal strain (RV GLS), were measured from the apical four-chamber view [10]. The dedicated AutoLA feature was used to automatically evaluate the LA strain from a single apical four-chamber view during the reservoir, conduit, and contraction phases, defined as the LA reservoir strain (LASr), conduit strain (LAScd), and contraction strain (LASct). Ventricular end-diastole was used as the zero-strain reference point (R–R gating). The example of strain analysis is shown in Figure 1. In patients in sinus rhythm, all three LA strain components were measured. In patients who were in AF during echocardiography acquisition, only LASr and LAScd were assessed, as LASct was not measured because of the absence of organized atrial contraction. In AF patients, LAScd corresponded to the negative value of LASr [10]. If needed, the LV, RV and LA endocardial borders were manually traced and edited according to existing recommendations [8,10]. Strain measurements were obtained from three consecutive cardiac cycles and averaged for analysis. The frame-rate of the images was 40–80 Hz.

LV diastolic function was graded according to the 2016 recommendations [9], applying algorithm for estimation of LV filling pressures and grading LV diastolic function in patients with depressed LVEFs and patients with myocardial disease and normal LVEF. Mitral inflow was used first: an E/A ratio ≤ 0.8 with peak E ≤ 50 cm/s was classified as grade 1, and an E/A ratio ≥ 2 as grade 3. In all remaining patients (E/A ≤ 0.8 with E > 50 cm/s, or E/A > 0.8 and <2), the three variables above were evaluated (E/e’ > 14, TR velocity > 2.8 m/s, and LA volume index (LAVI) > 34 mL/m^2^). When 2 of 3, or 3 of 3 were negative, grade 1 diastolic dysfunction was assigned. When 2 of 3, or 3 of 3 were positive, grade 2 diastolic dysfunction was assigned. When only two parameters were available, grade 1 was assigned if both were negative and grade 2 if both were positive; if the two parameters were discordant, the diastolic grade was considered indeterminate. The same algorithm was applied to obstructive and nonobstructive HCM patients. As no guideline-endorsed HCM-specific LASr thresholds for grading diastolic dysfunction are currently available, for exploratory analysis, patients were also categorized into LVDD grades using LASr thresholds, as proposed by Singh et al. (2017): ≥35% (grade 0), ≥24% to <35% (grade 1), ≥19% to <24% (grade 2), and <19% (grade 3) [11].

### 2.3. Statistical Analysis

Statistical analysis was performed using IBM SPSS Statistics, version 30.0 (IBM Corp., Armonk, NY, USA). Graphs were created using GraphPad Prism version 11.0.0 (GraphPad Software, Boston, MA, USA). The normality of continuous variables was assessed using the Shapiro–Wilk test. If variables were nonnormally distributed, nonparametric tests were used, and the data are presented as medians (interquartile ranges). Categorical variables were compared using the chi-square test. When more than 20% of the expected cell counts were <5, the Fisher–Freeman–Halton exact test (Monte Carlo method) was used instead. Continuous variables were compared using the Mann–Whitney U test (two groups) or the Kruskal–Wallis test (≥3 groups).

Agreement between LASr-based and conventional LV diastolic dysfunction grading was assessed using the weighted Cohen’s κ (kappa). Correlations between LA strain parameters and clinical/echocardiographic variables were assessed using Spearman’s rank correlation (r < 0.3—weak, 0.3 ≤ r ≤ 0.7—moderate, r > 0.7—strong). Logistic regression was used to evaluate associations between variables and AF. Multivariable models were adjusted for age, LAVI, and LV mass index (LVMI), which were selected as clinically relevant factors associated with AF and atrial remodeling. Sensitivity analyses were additionally adjusted for obstructive HCM phenotype. The results are presented as odds ratios (ORs) with 95% confidence intervals (CIs). Missing data were handled by analyzing only complete cases. A two-tailed *p* value < 0.05 was considered statistically significant.

## 3. Results

### 3.1. Baseline Characteristics

This study included 43 women and 66 men (median age 59 years [49.5–65.5]). Fifty (45.9%) patients were diagnosed with a nonobstructive phenotype, and 59 (54.1%) had obstructive HCM. Septal reduction therapy (myectomy or alcohol ablation) was performed in 24 patients (22%) after the initial echocardiographic examination. Pathogenic or likely pathogenic variants were identified in 40 (36.7%) patients, whereas 69 (63.3%) had variants of uncertain significance or no identifiable variants. Most patients with positive genetic test results were in grades 0 and 3, whereas the majority of patients without pathogenic variants had grade 2 and grade 3 LASr-based LVDD (Table 1).

Baseline clinical characteristics, comorbidities, and medical treatment are presented in Table 1. Age (*p* = 0.006) and BNP (*p* = 0.010) increased significantly across LASr-based LVDD severity (Table 1) and showed weak correlations with LA strain measurements (Table 2, *p* < 0.001). Arterial hypertension (80%) and dyslipidemia (67%) were the most frequent cardiovascular comorbidities among the whole cohort of patients. AF was documented in 32 (29%) patients, whereas only 3 had permanent AF (1 in each of grades 0–2). The remaining (*n* = 106) patients were in sinus rhythm during the echocardiographic examination. Most patients were symptomatic (NYHA I—20 (18.3%), II—64 (58.7%), III—25 (23.0%), IV—0 (0%)), received β-blockers (88%), angiotensin-converting-enzyme inhibitors or angiotensin II receptor blockers (64%), or statins (56%). A detailed analysis of the subgroups by LASr-based grades is shown in Table 1.

### 3.2. Diastolic Dysfunction Evaluation: Conventional Methods vs. LASr-Based Grading

LVDD was assessed using both conventional echocardiographic parameters and LASr-based grading. Thirteen patients had incomplete conventional LVDD evaluation data and were excluded from LVDD method comparison analysis, leaving 96 patients for this analysis. The distribution of LVDD grades differed significantly between methods (*p* = 0.010).

**Figure 2:**
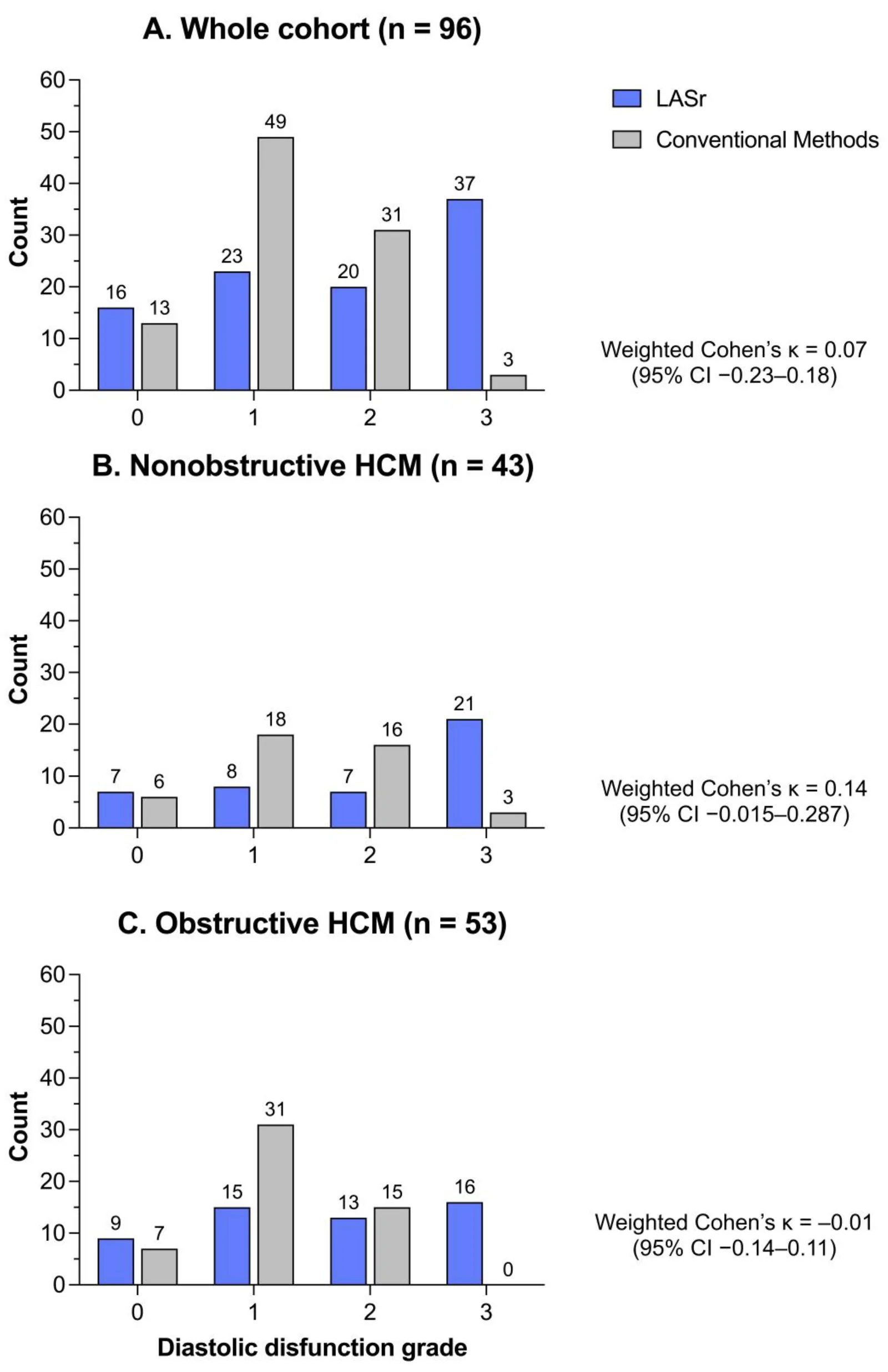
Distribution of diastolic dysfunction grades by conventional and LASr-based echocardiographic assessment in HCM. Bars represent number of patients in each diastolic dysfunction grade (0–3) assessed by conventional Doppler criteria and LASr-based grading. Figure (**A**) shows whole study population, Figure (**B**)—nonobstructive and (**C**)—obstructive HCM subgroup. Weighted Cohen’s κ values indicate agreement between grading methods. Abbreviations: HCM, Hypertrophic cardiomyopathy; LASr, left atrial reservoir strain, CI, confidence interval.

Across the whole study population, approximately 45% of patients were reclassified into higher LVDD categories when LASr-based evaluation was used compared with conventional methods. LASr-based grading revealed a substantially greater prevalence of advanced LVDD than did conventional criteria (grade 3: 37 (39%) vs. 3 (3%), *p* < 0.001; Figure 2A). In contrast, there was no significant difference between methods in the grade 0 assignment (17% vs. 14%, *p* = 0.273). Overall, the agreement between the two methods was poor (weighted κ = 0.07, 95% CI −0.23–0.18).

In patients with nonobstructive HCM (Figure 2B), the LVDD grade distribution was similar to that observed in the whole cohort, with a shift toward higher grades using LASr-based evaluation and poor intermethod agreement (weighted κ = 0.14, 95% CI −0.015–0.287). In obstructive HCM (Figure 2C), conventional echocardiographic assessment classified most (31 (58%)) patients as grade 1, with no patients assigned to Grade 3. In contrast, LASr-based grading markedly shifted classification toward more advanced LVDD, with 16 (30%) patients classified as grade 3, whereas the prevalence of grade 2 did not differ between methods. Overall, the agreement between methods in obstructive HCM patients remained poor (weighted κ = −0.01, 95% CI −0.14–0.11).

### 3.3. Clinical and Echocardiographic Characteristics Across Different LVDD Severities

LA structural parameters increased with increasing LVDD grade, with significant changes in LA diameter (*p* = 0.008), volume (*p* = 0.006), and volume index (*p* = 0.003) (Table 3). All three LA strain measurements showed significant correlations with these parameters (Table 2, *p* < 0.001). Overall, 75.7% of patients demonstrated LA dilation (LAVI > 34 mL/m^2^). Despite preserved LAVI (≤34 mL/m^2^), reduced LASr (<35%) was present in 88.5% of these patients. However, no significant difference in distribution between groups was observed (*p* = 0.282).

E/e′ differed significantly across LASr-based categories (*p* = 0.002), with the highest median value observed in grade 2. E/e′ also significantly correlated with LASr and LAScd (both *p* < 0.001). LAScd and LASct, which reflect the LA’s conduit and contractile functions during late diastole, progressively worsened with increasing LVDD (both *p* < 0.001).

Subtle biventricular deformation parameters, including the LV GLS (*p* < 0.001), RV GLS (*p* < 0.001), and RV FWLS (*p* = 0.003), decreased significantly with increasing LASr-based LVDD grade and were correlated with LA strain parameters (all *p* < 0.05). In contrast, the LVEF remained preserved (*p* = 0.093) but was correlated with the LASr and LASct (r = 0.256, *p* = 0.01 and r = −0.244, *p* = 0.05, respectively). The peak tricuspid annular systolic velocity (S′) (*p* = 0.04) and pulmonary artery systolic pressure (PASP) (*p* = 0.011) also differed significantly across increasing LVDD grades, whereas the tricuspid annular plane systolic excursion (TAPSE) remained stable (Table 2 and Table 3).

### 3.4. Associations Between LA Strain and AF

Although the prevalence of AF did not differ significantly across LASr-based LVDD grades (Table 1), LA strain values differed significantly between patients with documented AF and those without a history of AF (Table 4). In logistic regression analysis, LASr and LAScd were significantly associated with documented AF. Each 1 percentage-point increase in LASr was associated with 6% lower odds of AF (OR 0.94, 95% CI 0.90–0.98). In contrast, each 1 percentage-point increase in LAScd (i.e., a less negative value) was associated with 14% higher odds of AF (OR 1.14, 95% CI 1.05–1.24). Both associations remained significant after adjustment for age, LAVI, and the LVMI (Table 5). In sensitivity analyses, after additionally adjusting for obstructive HCM phenotype, the associations of both LASr and LAScd with documented AF remained almost unchanged (LASr: OR 0.95, 95% CI 0.90–0.99, *p* = 0.017; LAScd: OR 1.11, 95% CI 1.02–1.21, *p* = 0.015). LASct was not associated with AF in logistic regression analysis (Table 5), despite statistically significant differences between patients with and without documented AF (Table 4).

## 4. Discussion

Our study demonstrated that a novel diastolic function classification system based on LASr could provide additional information on diastolic dysfunction in patients with HCM. LASr-based grading revealed a different distribution of LVDD categories compared to traditional echocardiographic diastolic function evaluation and showed poor agreement with it. Higher LASr-based LVDD grades were associated with worse conventional echocardiographic diastolic parameters, biventricular deformation parameters, and higher natriuretic peptide levels. Moreover, impaired LA strain was independently associated with documented AF in our HCM population. In the absence of guideline-validated HCM-specific LASr thresholds, the Singh et al. cutoffs were used as a literature-based reference; therefore, the resulting categories should be interpreted as exploratory rather than definitive LVDD grades.

LVDD is a major pathophysiological mechanism of HCM, explains symptoms and diminished exercise capacity, and may lead to advanced HF; thus, accurate grading of LVDD is crucial for making appropriate therapeutic decisions. However, noninvasive assessment of LVDD in patients with HCM is still not well established and remains complex, as many standard echocardiographic parameters have a weak correlation with LV filling pressure [5,6]. The American Society of Echocardiography (ASE)/EACVI diastolic function guidelines recommend the use of the same algorithm for both HCM patients and the general population, including basic parameters such as e′, E/e′, TR velocity, and LAVI [9]. Many of these parameters are often discrepant, and obtaining all of them is sometimes impossible in HCM patients, making this classification challenging. The mitral e′ is abnormal in most patients with HCM, leading to a weak correlation between the E/e′ ratio and invasively measured LV filling pressure. Abnormally high E/A ratios should be interpreted with caution in young HCM patients, and normal pulmonary pressure (TR velocity < 2.5 m/s) should be helpful when differentiating grade 3 LVDD from normal filling pressure with an E/A ratio > 2.0 [6,9].

The new EACVI clinical consensus statement of multimodality imaging in HCM patients recommended LA strain, particularly LA reservoir strain, in the assessment of diastolic function in HCM when the four-criteria approach is inconclusive [5]. LA function decreases in the early stages of LVDD, whereas LA volume increases as a result of a chronic increase in LV filling pressure [12]. Several mechanisms contribute to increased LA afterload and complex atrial remodeling in patients with HCM, including LV remodeling leading to LVDD, MR, and LVOT obstruction. Changes in LA contractility, chamber architecture, and electrophysiology are defined as “atrial myopathy”, a novel marker of HCM disease severity and an independent prognostic factor for cardiovascular events. Speckle-tracking echocardiography is a valuable tool for the early detection of LA dysfunction, even when LA dimensions remain within normal limits [13]. In our study sample, 75.7% of patients demonstrated LA dilation (LAVI > 34 mL/m^2^); however, among patients with a normal LAVI (≤34 mL/m^2^), 88.5% still had a reduced LASr (<35%). Previous study has shown associations between LASr and invasively measured LV filling pressures, supporting its potential complementary role in diastolic assessment [14]. In our study, the distribution of LVDD grades differed significantly between conventional and LASr-based methods. Approximately 45% of patients were reclassified into higher diastolic dysfunction categories when LASr-based evaluation was used, with the most pronounced upward shift observed in the nonobstructive subgroup. However, this difference should be interpreted carefully, as invasive filling pressures were not available as a reference standard.

We observed significant associations between LASr and conventional diastolic indices, supporting the role of LA deformation as an integrative marker of diastolic dysfunction. LASr showed a moderate inverse correlation with E/e′ and LAVI, consistent with LV filling pressure and chronic atrial load. Both the LA conduit and contractile strain components were also significantly impaired and correlated with diastolic dysfunction parameters, supporting the potential value of comprehensive LA dysfunction evaluation in HCM. In addition, all LA strains correlated with LA diameter, confirming the findings of previous studies [15,16]. These findings align with those of previous studies, demonstrating that LA strain progressively decreases with worsening diastolic function, as defined by conventional criteria, and could aid in HF event prediction [6].

Decreasing LASr was significantly associated with worse LV deformation parameters, despite preserved LVEF. Lee et al. reported similar findings, as the LV GLS decreases with increasing LVDD, whereas the LVEF remains stable [6]. Another study suggested that LA strain may be equally predictive of HF progression along with LV GLS, therefore supporting broader implementation of atrial deformation measurements in clinical practice [15]. RV deformation parameters also worsened across LASr-based LVDD categories. RV GLS, RV FWLS, and tricuspid S′ all decreased significantly, whereas PASP increased, despite the unchanged TAPSE. These changes may reflect biventricular dysfunction involvement and increased pulmonary pressures. Previous studies have linked these findings to right ventricular–pulmonary arterial (RV–PA) uncoupling and HF progression [17,18]. Li et al. also reported the importance of RV evaluation in the HCM population and its role in AF prediction [19].

Our findings demonstrate an association between impaired LA strain values and AF. We found that each 1 percentage-point increase in LASr was associated with approximately 6% lower odds of AF, even after adjustment for age, LA size, and LVMI. Given the relatively small number of patients with documented AF, the multivariable models were intentionally kept parsimonious to reduce the risk of overfitting. Residual confounding by other HCM-related factors, such as MR, LVOT obstruction, cannot be excluded. However, additional adjustment for obstructive HCM phenotype did not change the associations of LASr or LAScd with documented AF. Our results align with other multiple studies, where reduced LA strain independently predicted new-onset AF, although these differ from ours in design. Debonnaire et al. demonstrated that a reduced LASr (≤23.4%) predicts new-onset AF independent of LA size, highlighting its sensitivity for early atrial dysfunction [20]. A meta-analysis by Hussain et al. reported that not only LA reservoir but also conduit, and booster strains were significantly lower in HCM patients than in controls and that reduced LA strain strongly predicted AF and MACE [7]. These were prospective analyses of AF-free individuals, whereas our cross-sectional analysis compares patients with and without a history of AF; our data are therefore concordant in direction but cannot replicate their predictive findings. Reduced LA strain likely reflects increased atrial fibrosis and impaired atrioventricular coupling, creating an arrhythmogenic substrate for AF [6,7,13]. Conversely, established AF itself promotes atrial fibrosis and contractile dysfunction, so the observed association is likely bidirectional. It is beneficial to incorporate LA strain measurements, as they reflect LA dysfunction, which is not fully captured by the LA diameter or volume increase [21].

In summary, our findings demonstrated that LA strain parameters, especially LASr, provide additional information for LVDD evaluation and atrial remodeling in HCM. The inclusion of these methods in initial echocardiographic evaluations of the HCM population results in a different distribution of diastolic dysfunction grades and provides insight into the atrial substrate associated with AF. However, prospective studies with invasive filling pressure measurements are needed to determine whether LA strain improves LVDD grading and can be used in risk stratification for incident AF.

### Study Limitations

This study has several limitations. First, it was a single-center retrospective cross-sectional study with a relatively small sample size, which may limit the generalizability of the findings and allows only the assessment of associations between parameters. Second, the study population was restricted to patients with HCM who had undergone genetic testing. As genetic testing is not required for the clinical diagnosis of HCM, this inclusion criterion may have introduced some degree of selection bias and may limit the generalizability of our findings to the broader HCM population. Because AF was assessed from medical history, we cannot establish whether impaired LA strain preceded AF or resulted partly from AF-related atrial remodeling. Accordingly, our results should be interpreted as an association between AF history and impaired LA reservoir function, and not as evidence that LA strain identifies patients at future risk of AF. Echocardiographic images were retrospectively obtained from our hospital database; therefore, some measurements were incomplete, or image quality was poor, resulting in missing data. In some patients, the apical two-chamber view was unavailable, limiting biplane LA evaluation. Intraobserver and interobserver reproducibility of LA strain measurements was not assessed. Although LA strain has demonstrated good reproducibility in previous studies, measurement variability and vendor/software dependence may have influenced strain values and classification, limiting generalizability. Furthermore, the LASr thresholds used for exploratory categorization were not specifically derived or validated in an HCM population. Our patients did not undergo cardiac catheterization; therefore, we could not determine whether LASr-based grading more accurately reflects LV filling pressures than conventional echocardiographic assessment. Future prospective studies should compare both approaches against invasively measured filling pressures.

Patients continue to undergo routine clinical follow-up at our center, and longitudinal outcome data are being collected for future analyses. However, larger independent prospective studies are also needed to validate the prognostic value of LASr in HCM patients.

## 5. Conclusions

LASr provides additional information on diastolic dysfunction in HCM patients and is associated with documented AF. LASr-based grading assigned more patients to advanced LVDD grades than conventional assessment. The observed association with AF requires prospective validation before LA strain can be used for risk stratification.

## Figures and Tables

**Table 1:** Clinical characteristics according to LASr-based left ventricular diastolic dysfunction.

Variable	Grade 0 (*n* = 20)	Grade 1 (*n* = 28)	Grade 2 (*n* = 22)	Grade 3 (*n* = 39)	*p* Value
Age, years	57.0 [50.3–64.0]	51.5 [42.5–60.0]	61.0 [53.5–71.8]	64.0 [50.0–69.0]	0.006
BNP, ng/L	70.7 [25.5–174.0]	69.4 [43.5–283.6]	81.4 [26.3–183.7]	179.8 [120.1–324.1]	0.010
BMI, kg/m^2^	28.6 [24.9–29.7]	27.0 [24.3–30.5]	28.7 [25.9–31.6]	30.2 [25.9–32.6]	0.105
Obstructive HCM	11 (55.0)	17 (60.7)	14 (63.6)	17 (43.6)	0.385
Pathogenic or likely pathogenic variants/VUS or no identifiable variants	17/3 (85.0/15.0)	8/20 (28.6/71.4)	1/21 (4.5/95.5)	14/25 (35.9/64.1)	<0.001
NYHA class (I/II/III/IV)	1/14/5/0 (5.0/70.0/25.0/0.0)	4/16/8/0 (14.3/57.1/28.6/0.0)	3/15/4/0 (13.6/68.2/18.2/0.0)	12/19/8/0 (30.8/48.7/20.5/0.0)	0.244
ICD	4 (20.0)	3 (10.7)	4 (18.2)	5 (12.8)	0.767
Pacemaker	3 (15.0)	5 (17.9)	1 (4.5)	4 (10.3)	0.478
Arterial hypertension	13 (65.0)	20 (71.4)	19 (86.4)	35 (89.7)	0.073
Dyslipidemia	12 (60.0)	16 (57.1)	14 (63.6)	31 (79.5)	0.209
Atrial fibrillation	4 (20.0)	9 (31.9)	7 (31.8)	12 (30.8)	0.789
Stroke	1 (5.0)	3 (10.7)	1 (4.5)	4 (10.3)	0.845
Diabetes mellitus	2 (10.0)	1 (3.6)	1 (4.5)	4 (10.3)	0.704
Chronic kidney disease (eGFR < 60 mL/min/1.73 m^2^)	2 (10.0)	4 (14.3)	3 (13.6)	2 (5.1)	0.546
Ventricular arrhythmias	5 (25.0)	9 (32.1)	4 (18.2)	11 (28.2)	0.725
Conduction abnormalities	8 (40.2)	4 (14.3)	2 (9.1)	8 (20.5)	0.084
Obesity (BMI > 30 kg/m^2^)	3 (15.0)	12 (42.9)	10 (45.5)	16 (41.0)	0.127
β-blocker use	18 (90.0)	24 (85.7)	20 (90.9)	34 (87.2)	0.978
ACE inhibitors/ARBs use	13 (65.0)	14 (50.0)	15 (68.2)	27 (69.2)	0.396
Mineralocorticoid receptor antagonist use	3 (15.0)	6 (21.4)	6 (27.3)	5 (12.8)	0.507
Sacubitril/Valsartan use	3 (15.0)	5 (17.9)	3 (13.6)	3 (7.7)	0.623
SGLT2 inhibitor use	5 (25.0)	2 (7.1)	2 (9.1)	2 (5.1)	0.124
Diuretic use	9 (45.0)	10 (35.7)	11 (50.0)	15 (38.5)	0.732
Calcium channel blocker use	4 (20.0)	5 (17.9)	7 (31.8)	5 (12.8)	0.353
Anticoagulant therapy	6 (30.0)	12 (42.9)	10 (45.5)	13 (33.3)	0.636
Antiplatelet therapy	4 (20.0)	5 (17.9)	3 (13.6)	10 (25.6)	0.745
Amiodarone use	1 (5.0)	2 (7.1)	2 (9.1)	4 (10.3)	0.967
Statin use	8 (40.0)	14 (50.0)	12 (54.5)	27 (69.2)	0.153

Data are presented as the median [interquartile range] or n (%). Abbreviations: LASr, left atrial reservoir strain; BNP, B-type natriuretic peptide; BMI, body mass index; VUS, variant of uncertain significance; HCM, hypertrophic cardiomyopathy; NYHA, New York Heart Association; ICD, implantable cardioverter defibrillator; eGFR, estimated glomerular filtration rate; ACE inhibitors, angiotensin-converting-enzyme inhibitors; ARB, angiotensin II receptor blocker; SGLT2, sodium–glucose cotransporter-2.

**Table 2:** Correlation of left atrial strain parameters with clinical and echocardiographic parameters.

Parameter	Correlation Coefficients		
	LASr	LAScd	LASct
Age, years	−0.262 **	0.359 **	−0.044
BNP, ng/L	−0.300 **	0.238 *	0.211 *
BMI, kg/m^2^	−0.243 *	0.305 **	0.086
LVMI, g/m^2^	−0.078	0.098	0.061
LA diameter, mm	−0.371 **	0.310 **	0.303 **
LA volume, mL	−0.350 **	0.304 **	0.274 **
LAVI, mL/m^2^	−0.352 **	0.303 **	0.243 *
E/e′	−0.363 **	0.475 **	0.104
LV GLS, %	−0.401 **	0.183	0.350 **
RV GLS, %	−0.404 **	0.274 **	0.271 **
RV FWLS, %	−0.318 **	0.246 *	0.178
LVEF, %	0.256 **	−0.085	−0.244 *
S′, cm/s	0.178	−0.038	−0.127
TAPSE, mm	−0.063	0.132	−0.209
PASP, mmHg	−0.372 **	0.216 *	0.220 *

* Correlation is significant at the 0.05 level (2-tailed). ** Correlation is significant at the 0.01 level (2-tailed). Abbreviations: LASr, left atrial reservoir strain; LAScd, left atrial conduit strain; LASct, left atrial contractile strain; BNP, B–type natriuretic peptide; BMI, body mass index; LVMI, left ventricular mass index; LA, left atrium; LAVI, left atrial volume index; E/e′, the ratio of mitral inflow early diastolic velocity to early diastolic mitral annular velocity; LV GLS, left ventricular global longitudinal strain; RV GLS, right ventricular global longitudinal strain; RV FWLS, right ventricular free wall longitudinal strain; LVEF, left ventricular ejection fraction; S′, peak systolic velocity at the tricuspid annulus; TAPSE, tricuspid annular plane systolic excursion; PASP, pulmonary artery systolic pressure. Spearman’s rank correlation was used for statistical analysis.

**Table 3:** Comparison of clinical and echocardiographic parameters across LASr-based LVDD Grades.

Variable	LASr-Based LVDD Grades				
	Grade 0 (*n* = 20)	Grade 1 (*n* = 28)	Grade 2 (*n* = 22)	Grade 3 (*n* = 39)	*p* Value
LA diameter, mm	42.0 [37.0–45.0]	41.0 [37.0–46.8]	42.5 [40.5–48.0]	47.0 [42.8–50.0]	0.008
LA volume, mL	75.9 [66.0–95.3]	77.9 [66.0–93.6]	88.3 [66.0–106.3]	103.0 [80.0–123.0]	0.006
LAVI, mL/m^2^	35.8 [32.3–48.6]	38.2 [33.0–48.5]	44.4 [31.1–48.7]	52.1 [40.3–59.3]	0.003
LASr, %	40.8 [36.9–43.4]	28.5 [25.6–31.0]	21.1 [20.0–22.0]	11.2 [9.3–14.8]	<0.001
LAScd, %	–19.5 [−26.2–(−15.1)]	–16.2 [−20.7–(−11.6)]	−9.7 [−12.7–(−7.4)]	−9.3 [−12.3–(−5.5)]	<0.001
LASct, %	−20.3 [−24.5–(−12.1)]	−11.7 [−16.1–(−8.0)]	−11.6 [−13.0–(−8.7)]	−3.4 [−7.4–(−1.0)]	<0.001
LVMI, g/m^2^	119.9 [108.5–143.3]	147.0 [111.0–189.1]	126.1 [108.5–154.1]	134.7 [113.3–157.3]	0.193
E/e′	8.2 [6.9–9.8]	9.7 [7.0–14.7]	14.4 [9.8–18.1]	12.4 [8.9–16.2]	0.002
LV GLS, %	−21.1 [−24.2–(−16.7)]	−16.3 [−19.4–(−13.8)]	−13.8 [−15.5–(−11.2)]	−12.1 [−17.0–(−9.2)]	<0.001
RV GLS, %	−17.55 [−20.4–(−14.0)]	−16.6 [−19.8–(−14.3)]	−15.7 [−19.3–(−10.0)]	−13.0 [−16.7–(−9.6)]	<0.001
RV FWLS, %	−26.8 [−32.1–(−19.0)]	−20.9 [−26.8–(−18.0)]	−17.4 [−24.3–(−12.2)]	−17.2 [−21.4–(−14.6)]	0.003
LVEF, %	60.0 [50.5–63.5]	59.4 [52.0–63.0]	55.9 [49.7–60.8]	53.5 [48.0–60.0]	0.093
S′, cm/s	12.0 [10.5–15]	13.8 [12.9–14.9]	12.5 [11.0–14.3]	12.0 [10.0–14.0]	0.040
TAPSE, mm	22.5 [18.75–24]	24.0 [18.8–27.0]	20.0 [18.2–33.5]	22.5 [14.1–25.3]	0.860
PASP, mmHg	32.0 [26.0–35.0]	31.5 [24.8–35.0]	33.0 [28.3–41.5]	35.5 [30.0–42.3]	0.011

Data are presented as the median [interquartile range]. Abbreviations: LVDD, left ventricular diastolic dysfunction; AF, atrial fibrillation; LA, left atrium; LV, left ventricle; LAVI, left atrial volume index; LASr, left atrial reservoir strain; LAScd, left atrial conduit strain; LASct, left atrial contractile strain; LVMI, left ventricular mass index; E/e′, the ratio of mitral inflow early diastolic velocity to early diastolic mitral annular velocity; LV GLS, left ventricular global longitudinal strain; RV GLS, right ventricular global longitudinal strain; RV FWLS, right ventricular free wall longitudinal strain; LVEF, left ventricular ejection fraction; S′, peak tricuspid annular systolic velocity; TAPSE, tricuspid annular plane systolic excursion; PASP, pulmonary artery systolic pressure. Kruskal–Wallis test was used for statistical analysis. LASct was not assessed in patients with AF during echocardiography; therefore, the sample sizes for LASct were: grade 0, *n* = 19; grade 1, *n* = 27; and grade 2, *n* = 21; grade 3, *n* = 39.

**Table 4:** Comparison of left atrial strain parameters between patients with and without documented AF.

LA Strain	Documented AF	No History of AF	*p* Value
LASr	15.7 [10.4–(21.2)]	25.9 [19.0–32.7]	<0.01
LAScd	−9.5 [−12.2–(−5.4)]	−14.4 [−19.6–(−9.0)]	<0.01
LASct	−6.8 [−11.7–(−1.8)]	−10.3 [−19.6–(−5.5)]	0.044

Data are presented as the median [interquartile range]. Abbreviations: AF, atrial fibrillation; LA, left atrium; LASr, left atrial reservoir strain; LAScd, left atrial conduit strain; LASct, left atrial contractile strain. LASct was measured only for patients in sinus rhythm during echocardiography.

**Table 5:** Univariable and multivariable binary logistic regression analyses of factors associated with atrial fibrillation.

Outcome	Model	Covariates Included	OR (95% CI) Per 1 Percentage-Point Increase in Signed Strain Value	*p* Value
LASr				
Documented AF	Univariable	None	0.94 (0.90–0.98)	0.003
	Multivariable	Age, LAVI, LVMI	0.95 (0.90–0.99)	0.014
LAScd				
Documented AF	Univariable	None	1.14 (1.05–1.24)	0.001
	Multivariable	Age, LAVI, LVMI	1.11 (1.02–1.21)	0.013
LASct				
Documented AF	Univariable	None	1.03 (0.98–1.10)	0.205
	Multivariable	Age, LAVI, LVMI	1.04 (0.98–1.10)	0.161

Abbreviations: AF, atrial fibrillation; LASr, left atrial reservoir strain; LAScd, left atrial conduit strain, LASct, left atrial contractile strain; LAVI, left atrial volume index; LVMI, left ventricular mass index; OR, odds ratio; CI, confidence interval. LAScd and LASct were entered into the regression models as signed values; therefore, an increase represents a less negative strain value.

## Data Availability

The raw data supporting the conclusions of this article will be made available by the authors on request.

## Abbreviations

The following abbreviations are used in this manuscript:

AF	Atrial fibrillation
HCM	Hypertrophic cardiomyopathy
HF	Heart failure
LA	Left atrium
LAScd	Left atrial conduit strain
LASct	Left atrial contractile strain
LASr	Left atrial reservoir strain
LAVI	Left atrial volume index
LV	Left ventricle
LV GLS	Left ventricular global longitudinal strain
LVDD	Left ventricular diastolic dysfunction
LVEF	Left ventricular ejection fraction
LVMI	Left ventricular mass index
LVOT	Left ventricular outflow tract
PASP	Pulmonary artery systolic pressure.
RV	Right ventricle
RV GLS	Right ventricular global longitudinal strain
RV FWLS	Right ventricular free wall longitudinal strain
SCD	Sudden cardiac death
